# *Bifidobacterium animalis* subsp. *lactis* and arginine mixture intake improves cognitive flexibility in mice

**DOI:** 10.3389/fnut.2023.1164809

**Published:** 2023-06-06

**Authors:** Kayo Ikuta, Daisuke Joho, Masaki Kakeyama, Mitsuharu Matsumoto

**Affiliations:** ^1^Dairy Science and Technology Institute, Kyodo Milk Industry Co., Ltd., Tokyo, Japan; ^2^Laboratory for Environmental Brain Science, Faculty of Human Sciences, Waseda University, Tokorozawa, Japan; ^3^Research Institute for Environmental Medical Sciences, Comprehensive Research Organization, Waseda University, Tokorozawa, Japan

**Keywords:** touchscreen operant system, microbiota-gut-brain axis, reversal learning, polyamines, functional food, learning-set

## Abstract

The relationship between intestinal microbiota and cognitive function has been investigated as one of the major topics within the intestinal microbiota–gut–brain axis. Although an increasing number of studies have demonstrated an improvement in learning and memory when using probiotics or prebiotics, to date, there are no studies that target the cognitive flexibility observed in the early stages of several neuropsychiatric diseases, including dementia. We have recently developed a novel behavioral task using the touchscreen operant system to assess cognitive flexibility. We found that the disruption of the intestinal microbiota in mice induced a decline in cognitive flexibility. In the present study, we investigated the effects of treatments consisting of *Bifidobacterium animalis* subsp. *lactis* and arginine (Bifal + Arg), which promote the production of intestinal bacterial polyamine, on cognitive flexibility in the mouse model. Male C57BL6 mice orally treated with Bifal + Arg three times a week gradually decreased the 1st-choice incorrect diagonal rate with repeated reversals compared with the control group. Furthermore, in serial reversal phases, Bifal + Arg-treated mice shifted to the behavior of choosing a new correct spot more quickly after the reversal, and this was faster with repeated reversals. These results indicate that this treatment adapts to change and improves cognitive flexibility. This is the first report to show that intestinal environmental control, including probiotics and prebiotics, improves cognitive flexibility in mice.

## 1. Introduction

Cognitive flexibility, a characteristic that is considered to be a part of executive functions, is the ability to organize appropriate goal-directed actions in an ever-changing environment. Difficulties in cognitive flexibility are observed in various neuropsychiatric diseases such as autism, attention deficit hyperactivity disorder, obsessive-compulsive disorder, schizophrenia, and dementia during all life stages (1–5). In particular, in the case of dementia, an impairment in cognitive flexibility appears during the early stages as a delay in adaptation to a changing environment, which may be recoverable; hence, cognitive flexibility may be considered a therapeutic target for preventing the onset and/or progression of dementia.

In previous studies using mice, such adaptation ability was analyzed using a simple reversal task in the Morris water maze (6, 7) and visual discrimination tests (8), in which correct and incorrect behaviors were switched. It was indicated that mice could not form a reversal learning-set, involving not only the discrimination reversal learning task but also the “learn to learn” reversal task, resulting in a small error rate after reversal task repetition, which is the case in humans, monkeys, birds, and rats (9–11). However, using IntelliCage (12, 13) and a touchscreen operant system (14), we reported in our previous studies that mice can also form a reversal learning-set after the repetition of behavioral sequencing-based reversal tasks. The cognitive flexibility test using a touchscreen operant system is new and, to date, it has few examples of applications. However, the cognitive flexibility test with a touchscreen operant system is similar to the IntelliCage task, which has been employed in many mouse studies (15). Accordingly, we applied the cognitive flexibility analysis task of IntelliCage to that of a touchscreen operant system with the aim of achieving higher probability of extrapolation to humans. During these tasks, mice were initially required to distinguish between rewarded and never-rewarded corners/spots and move between the two distantly positioned rewarded corners/spots. Then, serial reversals were introduced, in which diagonal spatial patterns of rewarded and never-rewarded corners/spots were switched repetitively. These methods could be used for the study of functional foods for diseases related to cognitive flexibility impairment such as dementia, autism, and schizophrenia. However, to date, no study has investigated the effects of functional foods, including probiotics and prebiotics, on impaired cognitive flexibility using these methods.

Recent studies have revealed that intestinal microbiota affects the gut-brain axis, which influences bidirectional signaling between the gastrointestinal tract and the brain via the nervous system and neurotransmitters, a concept termed as the “microbiota-gut-brain axis” (16). Cognitive dysfunction and reduced brain-derived neurotrophic factor expression in the hippocampus have been reported in germ-free mice and mice with antibiotic-induced dysbiosis (17–19). In addition, we reported that dysbiosis caused by antibiotic administration impairs cognitive flexibility in mice (14).

Polyamines are bioactive amines that are ubiquitously present in all cells and essential for maintaining cellular health (20–22). Although cellular polyamine homeostasis is maintained by synthesis and degradation enzymes in healthy young animals, polyamine production and concentration in tissues and organs, including the brain, decrease with age (23). However, polyamines can also be introduced exogenously with the diet or from the intestinal microbiota upon decreased cellular production. Oral spermidine administration has been shown to improve learning and memory in *Drosophila* (24, 25) and mice (26). Furthermore, we found that the combined administration of *Bifidobacterium animalis* subsp. *lactis* LKM512 and arginine (Bifal + Arg) promoted a stable production of intestinal bacteria-derived polyamines (27, 28) and that it improved spatial learning memory in aged mice in the Morris water maze test (27). However, the effects of Bifal + Arg on cognitive flexibility have not yet been reported. Thus, this study aimed to investigate the effects of Bifal + Arg administration on cognitive flexibility in young mice.

## 2. Materials and methods

### 2.1. Animals

This study is a randomized controlled trial conducted on the Bifal + Arg group and the control group of equal size with seven mice each. Male C57BL6/J mice (8 weeks old) were purchased from The Jackson Laboratories Japan, Inc. (Yokohama, Japan). All mice were housed in Micro Bio-Clean capsules (Tokiwa Kagaku Kikai Co., Ltd., Tokyo, Japan) with one mouse per cage under temperature-, humidity-, and light-controlled environment (25 ± 1°C, 50% humidity, 12-h light/dark cycle). The animals were bred on CRF-1 (Oriental Yeast Co., Tokyo, Japan) until the start of the study. The animal experiments were conducted in accordance with the protocols approved by the Kyodo Milk Industry Animal Use Committee (permit 2019-022).

### 2.2. Preparation and administration of Bifal + Arg

The mice were randomly divided into two groups (*n* = 7 per group), namely, control group and Bifal + Arg group. *Bifidobacterium animalis* subsp. *lactis* LKM512 was anaerobically pre-cultured [37°C for 48 h using Anaero Pack Kenki (Mitsubishi Gas Chemical Company, Inc., Tokyo, Japan)] on MRS agar medium (BD), and the colonies were used for the study. The growing colonies were collected with cotton swabs and suspended in a Dulbecco's phosphate-buffered saline (D-PBS) solution containing L-Arg (0.1 mg/g body weight), and the suspension was administered in the Bifal + Arg group. The mice were treated with D-PBS only in the control group. The solution was administered three times a week via oral gavage using a sonde from 9 days prior to the behavioral test until the end of the test.

### 2.3. Cognitive flexibility test

**Figure 1:**
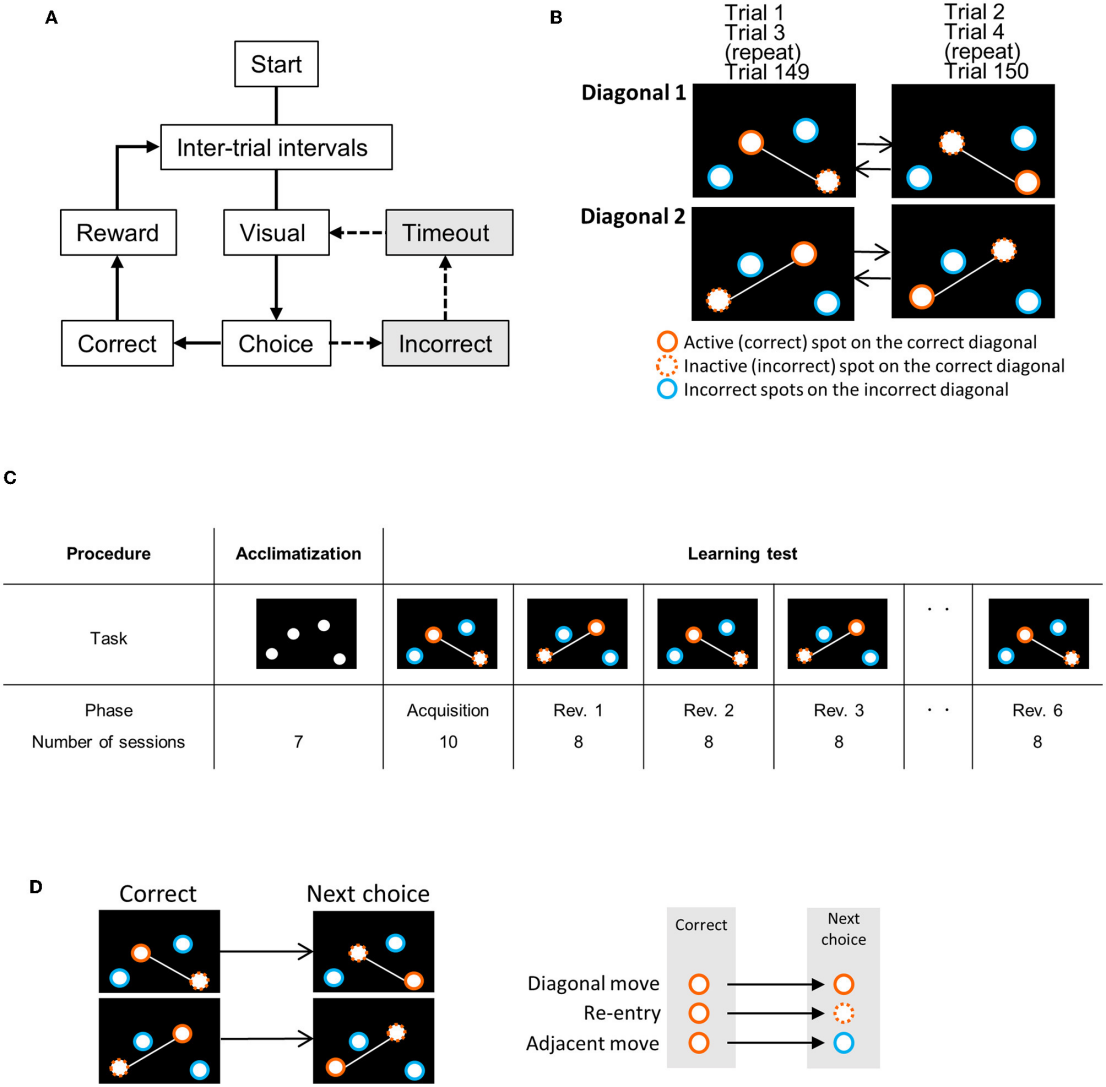
The experimental flowchart and correct/incorrect spots on the touchscreen in each phase. **(A)** Experimental flowchart. After the inter-trial interval, four spots were displayed on the touchscreen, and then, the trial began. When the mouse first chose (by nose-poking) the correct spot, it was rewarded and proceeded to the next trial. If the mouse chose incorrect spots, the touchscreen displayed the four spots again after a timeout. The trial continued until the mouse chooses the correct spot (dotted line). **(B)** Behavioral sequencing task: each spot on the correct diagonal (white line) is repeatedly changed from an active (correct) spot (orange circles) to an inactive (incorrect) spot (orange dotted line circle) in every trial. This session of this exchange is repeated until 150 trials are completed. The two spots on the other diagonal line (blue circle) are incorrect diagonal spots. **(C)** Experimental procedure. Acclimatization took 7 days. Reversal learning: the mice were rewarded only when they choose the active spot on the correct diagonal line, for example, diagonal 1. In the next phase, the correct diagonal line was replaced by the other line (diagonal 2), which was the previously incorrect diagonal line. In the first acquisition phase, mice performed at diagonal 1 (or 2) for 10 sessions, and in the next Reversal 1 (Rev. 1) and mice performed at diagonal 2 (or 1) for 8 sessions. The exchange of the correct diagonal lines was repeated every eight sessions. Whenever mice performed the acquisition phase on diagonal 1, they performed it on diagonal 2 in Rev. 1, 3, and 5, and then again back on diagonal 1 in Rev. 2, 4, and 6. Diagonal 1 and diagonal 2 were alternated to evaluate the behavior of reversal. **(D)** The definition of the first choice behavior pattern following correct spot choice. “Diagonal move” indicates a move from an active spot to the next active spot in the correct diagonal. “Re-entry” indicates a move from an active spot to the next inactive spot (previous active spot) in the correct diagonal. “Adjacent move” indicates a move from an active spot to an incorrect diagonal spot.

The cognitive flexibility test was performed using a touchscreen operant apparatus modified from a previously reported method (14). The flowchart and definition of movements on the touchscreen are shown in Figure 1. On the touchscreen, four holes were placed asymmetrically on the left, right, top, and bottom. The test summary is as follows:

Acclimatization: The mice were habituated to the touchscreen apparatus and the pellet dispenser (1 day) and then trained to choose a spot by the nose-poking behavior into the hole (6 days).

Behavioral sequencing task: After habituation, the behavioral sequencing task was conducted. In this task, the mice had to distinguish correct and incorrect diagonals and active and inactive spots on the correct diagonal. At the start of each trial, four spots on the touchscreen were displayed. The mice were rewarded when they chose an active spot on the correct diagonal, followed by the next trial. The four spots were displayed again after a timeout when the mice chose an incorrect spot (spots on the incorrect diagonal or inactive spots on the correct diagonal). The trial continued until the mouse chose the active spot and was rewarded. After the reward, the active and inactive spots were reversed for the next trial. This behavioral sequencing task was performed for 10 sessions as an acquisition phase. Each session consisted of 150 trials and lasted for 60 min.

Serial reversal learning: In the serial reversal learning using the behavior sequencing task, correct and incorrect diagonals were repeatedly reversed six times. Each reversal learning phase lasted for eight sessions.

Behavioral scores: The behavior of each session was assessed using the following behavioral scores (Supplementary Table S1): [1] “1st-choice incorrect diagonal rate”: percentage of choosing incorrect diagonal spots at the first choice of the trial. We defined it as the “1st-choice never rewarded rate” in our previous reports (14). [2] The rates of three choosing patterns a following correct spot choice during the acquisition phase (1st-choice behavior analysis): “diagonal move” (the first choice in a trial: move diagonally from the active spot to the next active spot), “adjacent move” (the first choice in a trial: move from the active spot to either of the two spots in the incorrect diagonal), and “re-entry move” (the first choice in a trial, nose-poke to the same spot). [3] “Cumulative diagonal correct move count” and “cumulative diagonal error move count”: Two consecutive choices on the correct diagonal were counted as the “diagonal correct move,” and two consecutive choices on the incorrect diagonal were counted as the “diagonal error move.” [4] “Diagonal correct move rate”: the rates of diagonal correct move in every 20-choice block of the first 100 choices in the first session of Rev. 5.

### 2.4. Statistics

All statistical analyses for finding differences in behavioral scores were performed using SPSS Statistics version 22 (IBM Corp, Armonk, NY, USA). The behavioral data were compared using a two-way repeated measure ANOVA using the Bonferroni correction. Error bars indicate the standard error of the mean. Statistical significance was set at a *p*-value of <0.05.

## 3. Results

### 3.1. Behavioral sequencing task

**Figure 2:**
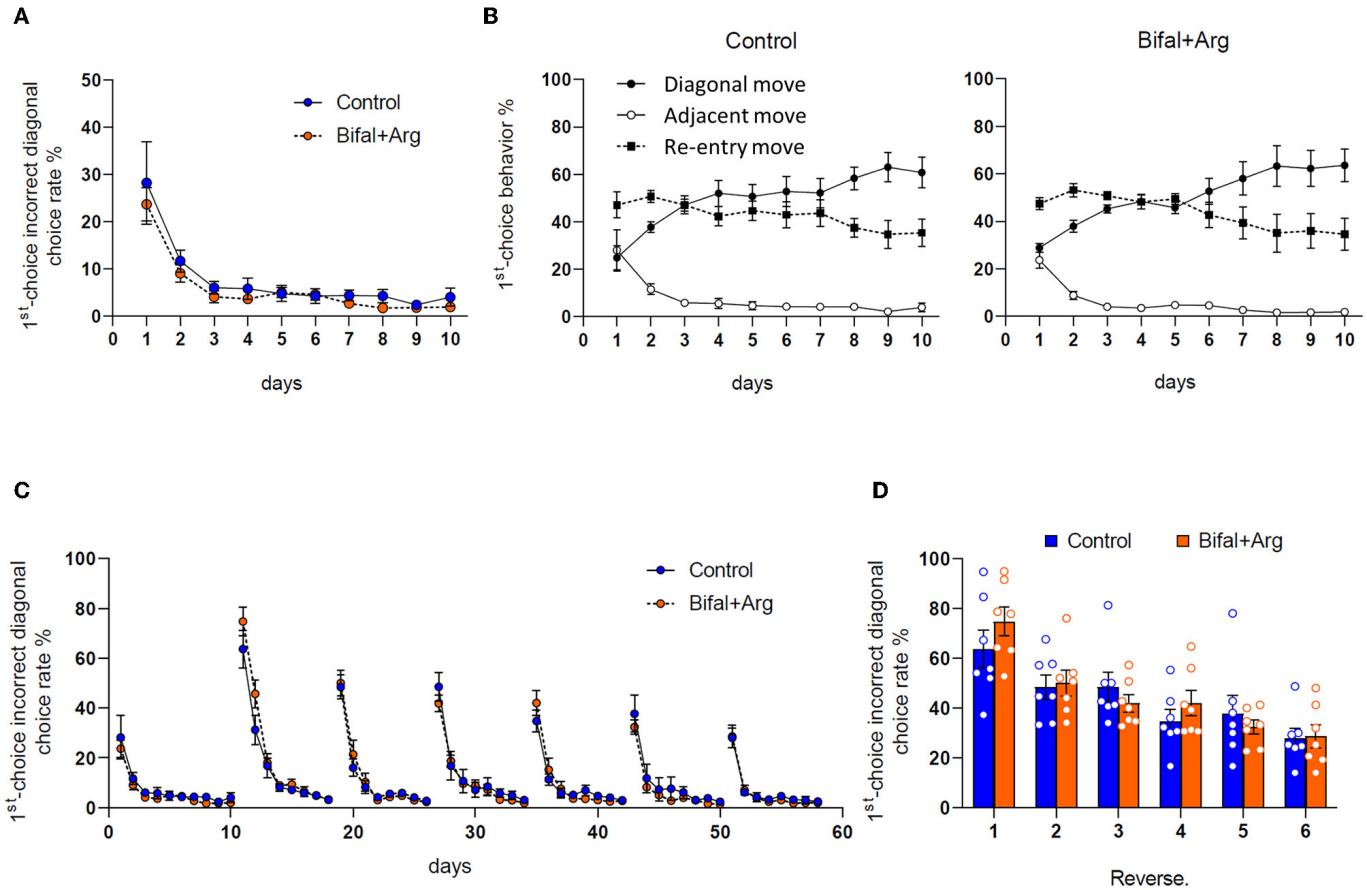
Effect of Bifal + Arg administration on the behaviors in the cognitive flexibility test. All data represent the mean and the standard error of the mean (SEM; *n* = 7 per group). **(A)** The rates of the incorrect diagonal spots choice at the first choice (blue circles in Figure 1) of the first 100 trials per session during the behavioral sequencing task. **(B)** The rates of the three move patterns [diagonal move (correct spot to correct spot); adjacent move (correct spot to incorrect diagonal spot); re-entry (correct spot to same spot)] in two consecutive choices during the behavioral sequencing task phase. **(C)** Change in the rates of the incorrect diagonal spots choice at the first choice during the test period. **(D)** Comparison of the rates of the incorrect diagonal spots choice at the first choice in the first session of each reversal phase.

The acquisition level of the behavioral sequencing task was assessed. The 1st-choice incorrect diagonal rate in the first session of the acquisition phase was 28.2 ± 8.7% in the control group and 23.7 ± 3.5% in the Bifal + Arg group, and these values decreased to <15% in the second session and <5% after ten sessions in both groups, with no differences between groups (Figure 2A). In the 1st-choice behavioral analysis (Figure 2B), the diagonal and adjacent move rates were comparable in both groups in the first session. However, diagonal move rates increased after the second session, and the adjacent move rates decreased. After 10 sessions, the adjacent move rates were under 5%, and the diagonal move rates were over 60%. The re-entry move rates were ~50% in the first session but decreased to ~35% after 10 sessions, with no differences between groups, as well as in the acquisition levels of this process, showing that both groups have a good understanding of the rules of the behavioral sequencing task and that the mice in both groups are ready for the serial reversal learning to analyze cognitive flexibility.

### 3.2. Adaptation to serial reversal learning

The 1st-choice incorrect diagonal rate during the cognitive flexibility test was analyzed. In the first session of Reversal 1 (Rev. 1), the 1st-choice incorrect diagonal rate was the highest during the test period, with 63.7 ± 7.6% in the control group and 74.8 ± 5.8% in the Bifal + Arg group (*p* = 0.27). In the second session, it decreased in both groups and was around the chance level of 50% (control group: 48.4 ± 4.9%, Bifal + Arg group: 50 ± 5.1%; Figure 2C). After Rev. 2, the 1st-choice incorrect diagonal rate in the first session gradually decreased, and in Rev. 3, the scores of both groups were below the chance level of 50% in the first session (control group: 48.6 ± 5.8%, Bifal + Arg group: 41.9 ± 3.5%, *p* = 0.35). The values of the 1st-choice incorrect diagonal rate in the first session after reversals were extracted (Figure 2D). In the Bifal + Arg group, this rate gradually decreased with repeated reversals, while in the control group, the 1st-choice incorrect diagonal rate increased slightly in Rev. 5 compared to that in Rev. 4.

### 3.3. Analysis of the first 200 choices in the first session of each reversal

**Figure 3:**
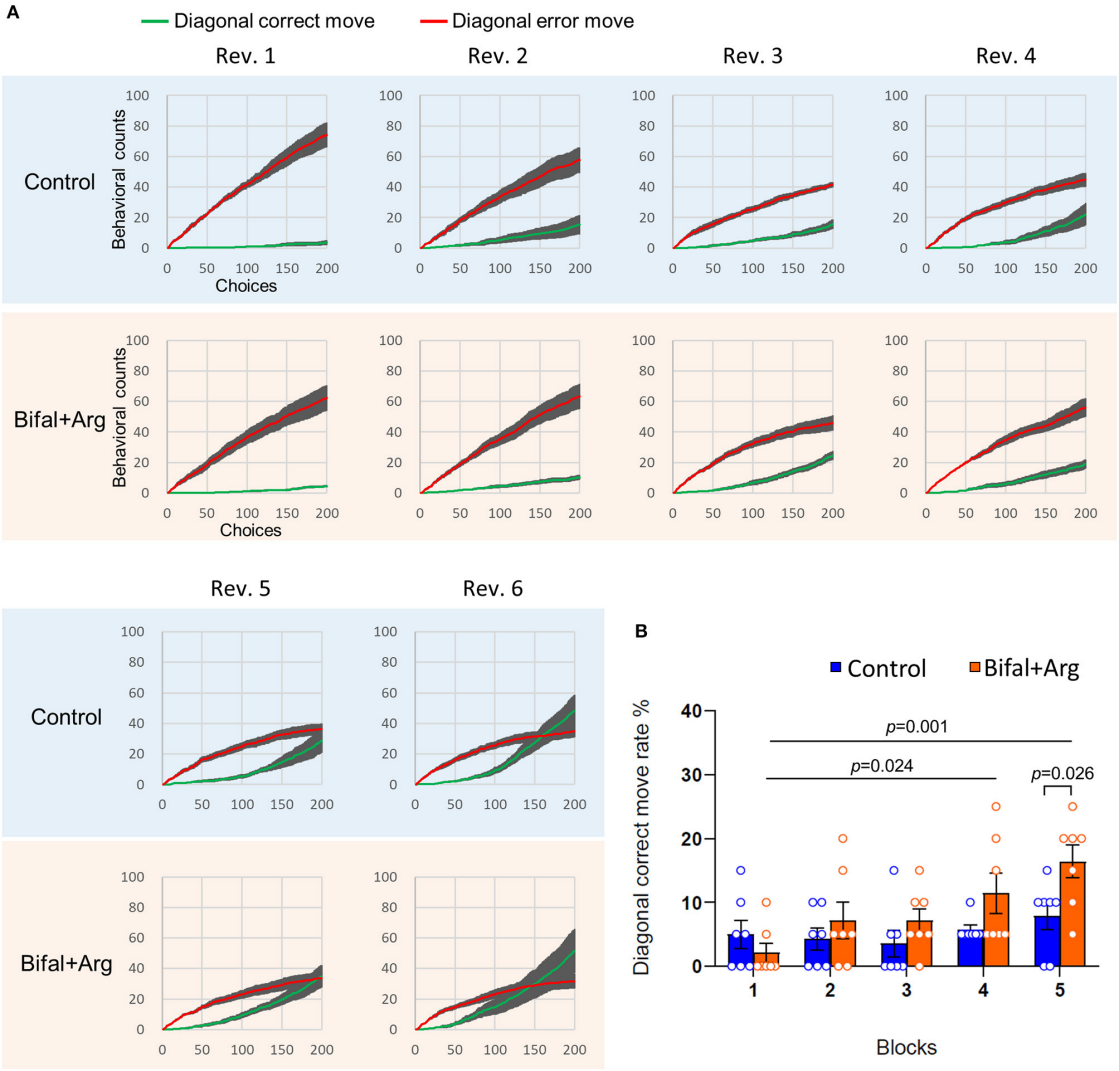
Comparison of adaptation speed to reversal contingency. **(A)** Cumulative diagonal correct move (green) and error move (red) counts within the first 200 choices of the first session of each reversal. **(B)** Diagonal correct move rate in every 20-choice block of the first 100 choices in the first session of Rev. 5.

We analyzed the behavior of the first 200 choices in the first session of each reversal to assess the choice behavior immediately after the reversal in detail. The cumulative count of consecutive choices of the two correct diagonal spots (cumulative diagonal correct move) together with the cumulative count of consecutive choices of the two incorrect diagonal spots (correct diagonal behavior in the previous reversal; cumulative diagonal error move) were analyzed (Figure 3A). The temporal increase dynamics of the cumulative diagonal correct move and error move counts were compared. There were no differences in these cumulative counts between the groups from Rev. 1 to Rev. 4. In Rev. 5, the increase in the cumulative diagonal correct move count was observed around the 150th choice in the control group. In contrast, in the Bifal + Arg group, an earlier increase was observed around the 100th choice. In addition, the cumulative diagonal correct move count was higher than the cumulative diagonal error move count before the 200th choice in the Bifal + Arg group. However, this count was consistently lower than the cumulative diagonal error move count in the control group. In Rev. 6, the control group showed an increase in the cumulative diagonal correct move count around the 100th choice, but the Bifal + Arg group showed an earlier increase around the 50th choice (see reference values of cumulative diagonal correct move and error move at the 50th, 100th, and 200th choices shown in Supplementary Table S2). These results indicate that adaptation to reversals was faster in the Bifal + Arg group than in the control group.

To precisely clarify the difference in the speed of recognizing the reversal rule, the diagonal correct move rates of the first 100 choices compiled for every 20-choice block were analyzed in the first session of Rev. 5 (Figure 3B). The diagonal correct move rate of the Bifal + Arg group was significantly higher than that of the control group in Block 5 (choices 81–100) in Rev. 5 (*p* = 0.026). Furthermore, in the Bifal + Arg group, the diagonal correct move rate gradually increased, and, in Block 4 (choices 61–80, *p* = 0.024) and Block 5 (choices 81–100, *p* = 0.001), it was significantly higher than that in Block 1 (choices 1–20) in Rev. 5. In contrast, a gradual and significant increase in the diagonal correct move rate was not observed in the control group.

## 4. Discussion

Recent studies have revealed that intestinal microbiota affect brain functions via the intestinal microbiota-gut-brain axis; hence, intestinal microbiota has been proposed as a target for the prevention and treatment of brain-related diseases (29–32). Dysbiosis occurs in patients with dementia, and probiotic administration improves cognitive function (33–35). However, few studies have targeted cognitive flexibility, which is impaired in the initial stages of dementia.

Previous cognitive flexibility tests in mice were performed using the Morris water maze test and the visual discrimination reversal task, which are very simple. The Morris water maze is a spatial learning test motivated by pain avoidance behavior toward water; hence, it is not suitable for extrapolation to humans. However, our cognitive flexibility test based on the performance determined using the touchscreen operant apparatus, which rewards motivation for learning, is similar to the Brixton spatial anticipation test used as a clinical assessment method of human executive function and thus is highly extrapolatable to humans (14). This test can estimate the animal reversal learning-set, indicating that animals can learn to learn (the formation of a learning-set). The concept of learning-set refers to the ability to acquire progressive shifts from trial-and-error to the immediate solving of new problems based on previously learned experiences. It is considered an executive function of the brain. We demonstrated that mice form a reversal learning-set by adapting their behavior more quickly to a change from previous experiences through repeated learning (12, 14). Bifal + Arg-treated mice switched to the new diagonal correct move after reversal faster than the control group. In addition, only Bifal + Arg-treated mice showed a gradually decreasing 1st-choice incorrect diagonal rate in the first session in each stage, indicating that Bifal + Arg promotes the formation of reversal learning-set and induces the early onset of the optimal behavior upon a change from past experiences through repeated reversal tasks, that is, the enhanced cognitive flexibility of mice. This is the first report to demonstrate the effects of intestinal microbial control, such as probiotics, prebiotics, and synbiotics, on cognitive flexibility.

The present results clearly demonstrate the possibility of enhanced cognitive flexibility with the administration of Bifal + Arg in mice. However, the detailed underlying mechanism remains unclear. Several reports have shown a relationship between polyamines and brain function. For example, polyamines are stored in astrocytes and neuronal vesicles in the brain and are involved in neurotransmitter secretion (36–38). Polyamines modulate memory consolidation by interacting with the polyamine-binding site on *N*-methyl-D-aspartate (NMDA) receptors associated with memory and learning (39). In *Drosophila*, oral polyamine (spermidine) supplementation improves age-dependent memory impairment by enhancing autophagy, neuroprotection, and synaptic plasticity (24, 25). In aged mice, dietary polyamine (spermidine) supplementation is transported to brain tissue, thereby improving cognition through increased hippocampal eIF5a hypusination and mitochondrial respiratory competence (26).

Furthermore, we have previously reported that intestinal polyamine production using Bifal + Arg improved spatial learning and memory in aged mice using the Morris water maze (27). Based on these findings, together with the results of the present study, we speculate that Bifal + Arg may influence brain function through the intestinal microbiota-gut-brain axis *via* polyamines. We also found that antibiotic-induced dysbiosis impaired cognitive flexibility in mice using the same test methods, suggesting that metabolite-derived microbiota, including polyamines, are probably involved in cognitive flexibility (14). Although the polyamine synthesis capacity of tissue declines with age and the tissue polyamine concentration decreases (23, 40), the polyamine level in the body is well maintained in the young mice used in this study. A few studies showed that the excessive administration of exogenous polyamine induces emaciation, aggressive behavior, and convulsion (41). However, the intercellular polyamine level is strictly regulated at an optimal concentration by the polyamine generation/degradation pathway in healthy mammals (21). Hence, healthy mice are unlikely to be affected by excessive polyamine dosage. In fact, our previous study demonstrated that Bifal + Arg administration for ~1 year promoted longevity without adverse effects (27). Therefore, intestinal microbiota-derived polyamines may undergo a different mechanism of cognitive flexibility than through the hippocampal eIF5a hypusination and mitochondrial respiratory competence described above. Further investigation is required to elucidate the mechanism underlying the microbiota-derived polyamine-gut-brain axis.

In this study, each mouse (9–23 weeks old) was independently bred in a separate cage for dietary restriction, maintaining their reward motivation. Several reports indicate that social isolation in separate cages affects the formation of sociality and learning memory (42–44), suggesting that our results were influenced by social isolation. However, since the greatest impact of social isolation occurs after weaning to 8 weeks old, when our mice were group-housed, and when other studies have shown results inconsistent with these studies, the impact of social isolation on this study appears to be limited.

There is a limitation to this study. The significance of the parallel administration of Bifal and Arg is unclear. The mixture of Bifal and Arg was administered because Bifal + Arg produced polyamines very efficiently in the previous study (27), but it is required for single administration of each solution in order to clarify the significance of parallel administration in the near future.

We conclude that the administration of Bifal + Arg improves cognitive flexibility. This study provides the first evidence that intestinal environmental control using functional food can improve cognitive flexibility.

Since impairment of cognitive flexibility occurs not only in the early stages of dementia but also in mental disorders that can develop at any stage, these findings may be applicable to other mental disorders in the future.

## Data availability statement

The original contributions presented in the study are included in the article/Supplementary material, further inquiries can be directed to the corresponding authors.

## Ethics statement

The animal study was reviewed and approved by Kyodo Milk Industry Animal Use Committee (permit 2019-022).

## Author contributions

KI and MM designed the study and created the figures. KI performed the experiments, statistically analyzed the data, and wrote the manuscript draft. MM supported the experiments. KI, DJ, MK, and MM interpreted the data. DJ revised the manuscript. MK and MM critically revised the manuscript. All authors contributed to the article and approved the submitted version.
