# Supplementary material for: Bifidobacterium animalis subsp. lactis and arginine mixture intake improves cognitive flexibility in mice
Source: Front Nutr. 2023 Jun 6;10:1164809. doi: 10.3389/fnut.2023.1164809 (PMC10279864; doi:10.3389/fnut.2023.1164809)
Supplement: Supplementary file 1 [file Data_Sheet_1.pdf]

Supplementary Table S1. Scoring system of the behaviors

Scoring items	Behaviors	Data presented in
1st-choice incorrect diagonal	Choose (nose-poke) to incorrect spots, on the incorrect diagonal line at the first choice of the trial	Fig. 2A, C, D
Diagonal move	Diagonal move from the active spot to next active spot on the correct diagonal line	Fig. 2B
Adjacent move	Adjacent move from the active spot on the correct diagonal line to either of the two spots on the incorrect diagonal line	Fig. 2B
Re-entry move	Choose (nose-poke) to the same spot consecutively (Move from the active spot to the next inactive spot on the correct diagonal line)	Fig. 2B
Diagonal correct move	Two consecutive choices (nose-pokes) on correct diagonal line	Fig. 3
Diagonal error move	Two consecutive choices (nose-pokes) on incorrect diagonal line	Fig. 3

Supplementary Table S2. The cumulative diagonal correct move counts at the 50th, 100th, and 200th choice after the reversal in first session of each reversal

	Rev. 1-1	Rev. 2-1	Rev. 3-1	Rev. 4-1	Rev. 5-1	Rev. 6-1
**The first 50 choices**						
Control	0.57±0.29	1.85±0.73	1.71±0.89	0.85±0.40	2.14±0.88	2.14±0.63
Bifal+Arg	0.28±0.18	2.00±0.53	1.71±0.68	1.71±0.47	2.42±0.94	3.85±1.20
**The first 100 choices**						
	Rev. 1-1	Rev. 2-1	Rev. 3-1	Rev. 4-1	Rev. 5-1	Rev. 6-1
Control	1.00±0.53	5.28±2.21	4.85±0.93	4.00±1.51	5.28±1.35	9.28±2.27
Bifal+Arg	1.14±0.55	4.57±1.17	6.85±1.72	6.00±1.57	9.42±1.92	14.71±5.02
**The first 200 choices**						
	Rev. 1-1	Rev. 2-1	Rev. 3-1	Rev. 4-1	Rev. 5-1	Rev. 6-1
Control	3.57±1.50	15.28±6.23	15.71±3.22	22.14±7.37	28.71±8.03	48.28±10.59
Bifal+Arg	4.42±0.71	10.28±1.61	24.57±2.93	19.00±3.11	34.85±7.48	51.57±14.49

Mean ± SEM
